# “One Health” Perspective on Prevalence of ESKAPE Pathogens in Africa: A Systematic Review and Meta-Analysis

**DOI:** 10.3390/pathogens13090787

**Published:** 2024-09-12

**Authors:** Ntelekwane George Khasapane, Sebolelo Jane Nkhebenyane, Kgaugelo Lekota, Oriel Thekisoe, Tsepo Ramatla

**Affiliations:** 1 Centre for Applied Food Safety and Biotechnology, Department of Life Sciences, Central University of Technology, 1 Park Road, Bloemfontein 9300, South Africa; snkhebena@ut.ac.za (S.J.N.); tramatla@cut.ac.za (T.R.); 2 Unit for Environmental Sciences and Management, North-West University, Potchefstroom 2531, South Africa; lekotaa.lekota@nwu.ac.za (K.L.); oriela.thekisoe@nwu.ac.za (O.T.)

**Keywords:** ESKAPE, pathogens, systematic review, meta-analysis, prevalence, Africa

## Abstract

The leading cause of hospital-acquired infections worldwide includes *Enterococcus faecium*, *Staphylococcus aureus*, *Klebsiella pneumoniae*, *Acinetobacter baumannii*, *Pseudomonas aeruginosa*, and *Enterobacter* spp. (ESKAPE) infections. These bacteria are commonly isolated from clinical settings and linked to a number of potentially fatal diseases associated with hospitals. The objective of this study was to review the prevalence of ESKAPE pathogens in Africa. We gathered and systematically reviewed the literature concerning the prevalence of ESKAPE pathogens, published in the English language from January 2014 to February 2024, from three databases (PubMed, Web of Science and ScienceDirect). Our overall results revealed that *S. aureus* was the most prevalent species (79.5%), followed by *A. baumannii* (27.6%), *K. pneumoniae* (24.2%), *Enterobacter* spp. (20%), *P. aeruginosa* (9.0%), and *E. faecium* (5.1%). Moreover, stool samples had the highest Pooled Prevalence Estimates (PPEs) of 44.0%, followed by urine, nasal, and blood samples with 37.3%, 26.9%, and 22.9%, respectively. For the diagnostic method used to identify these ESKAPE pathogens, VITEK-MS had the highest PPE of 55.2%, followed by whole genome sequencing and PCR with 37.1% and 33.2%, respectively. The highest PPE of ESKAPE pathogens was recorded in West Africa with 77.3%, followed by Central/Middle Africa and East Africa with 43.5% and 25.1%, respectively. The overall PPE of ESKAPE pathogens from humans, animals, the environment (water, soil, and surfaces) and food sources was 35.8%, 37.3%, 47.7%, and 34.2%, respectively. Despite their prevalence in nosocomial settings, studies have shown that the ESKAPE pathogens may be isolated from a range of environmental reservoirs, including soil, dumping sites, beach sand, wastewater, food, and fish farms, among others. This wide source of ESKAPE pathogens substrates indicates the need for a multidisciplinary collaborative partnership for epidemiological studies and intervention efforts by the human, veterinary, and environmental health sectors in Africa.

## 1. Introduction

The Centers for Disease Control and Prevention (CDC) declared that the ESKAPE bacteria are a serious worldwide health concern because they can increase morbidity and mortality when they infect people [1]. These germs have the potential to increase hospital stays and expenses for both patients and the healthcare system [2]. In 2008, the abbreviation ESKAPE was proposed, which refers to the class of pathogens that can “escape” the biocidal effect of antibiotics [3]. These pathogens include the *Enterococcus faecium*, *Staphylococcus aureus*, *Klebsiella pneumoniae*, *Acinetobacter baumannii*, *Pseudomonas aeruginosa*, and *Enterobacter* species. These bacteria are among the most common causes of infections that can be fatal, and it is well recognized that they can develop and spread antibiotic resistance [4,5]. In order to direct the development of new antibiotics, the World Health Organization (WHO) recently created a global priority list of pathogens resistant to antibiotics. Among the critical priorities is the Enterobacterales family, which is composed of some antibiotic-resistant organisms (e.g., third-generation cephalosporin-resistant Enterobacterales) [6,7]. Hence, the entire Enterobacterales group is frequently included in publications under the ESKAPE group of bacteria [8].

Compared to human medicine, there is a dearth of data regarding the prevalence of healthcare-associated infections (HCAIs) in veterinary medicine, despite a recent increase in awareness of the issue [9]. Furthermore, despite the growing significance of animal HCAIs in contemporary veterinary practice, infection control is still in its infancy [10]. This looks to be especially the case for companion animals, such as dogs, cats, and horses, where an increasing amount of research has documented the nosocomial outbreaks of various etiologies that are frequently linked to and made more difficult by the zoonotic and antibiotic-resistant characteristics of the microorganisms in question [10].

The ESKAPE pathogens are most likely present in the environment because of a variety of human activities, including swimming and bathing, improper disposal of hospital waste, sewage spills, and agricultural waste disposal [11,12]. Furthermore, ESKAPE pathogens have been found in beach sand and intertidal beach water [13]; industrial and municipal wastewater systems [14]; soils affected by human activity and dumping sites [11]; vegetables [15]; raw or prepared foods [16]; irrigation water, groundwater, and surface water systems, including drinking water systems [15]. Therefore, it is for the aforementioned reasons that the aim of this systematic review and meta-analysis was to review the continental prevalence of ESKAPE pathogens in humans, animals, and the environment (including food) in Africa.

## 2. Materials and Methods

### 2.1. Study Design, Systematic Review Protocol, and Registration

**Figure 1:**
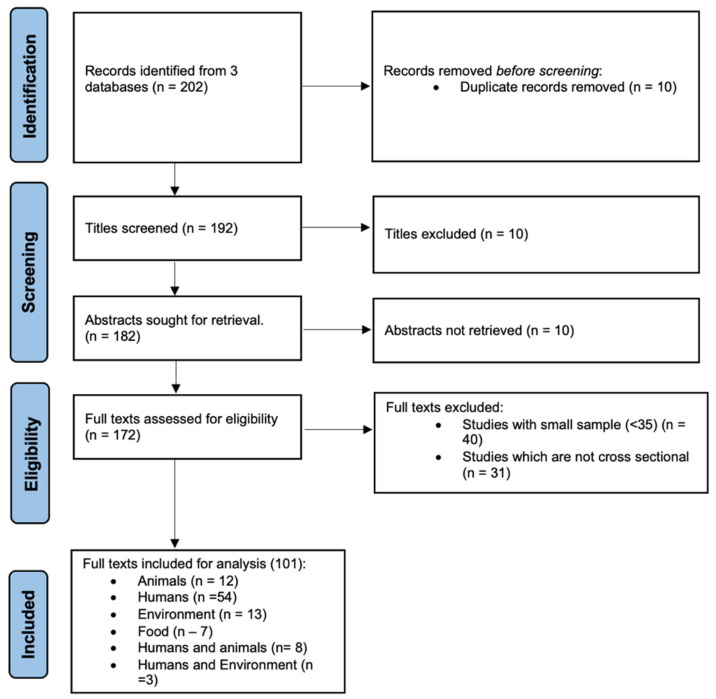
PRISMA flow diagram demonstrating the search and selection process of included studies in this review.

The Preferred Reporting Items for Systematic Reviews and Meta-analyses (PRISMA) guidelines [17] were followed in conducting this systematic review and meta-analysis (Figure 1). Data extraction, screening, and analysis involved searching through database systems for potentially relevant articles, determining their relevancy, and determining whether or not they were appropriate for inclusion in the review. The study is registered at the International Prospective Register of Systematic Reviews (PROSPERO): CRD42024579291.

### 2.2. Search Strategy

The following word strings were used in the methodical literature search: prevalence AND *Acinetobacter baumannii* AND *Pseudomonas aeruginosa* AND *Enterobacter* species, AND *Staphylococcus aureus* AND *Klebsiella pneumoniae* AND *Enterobacter* spp. AND Africa. The combination of terms mentioned above was used to search the bibliographic databases—PubMed (https://pubmed.ncbi.nlm.nih.gov/, accessed on 9 September 2024), ScienceDirect (https://www.sciencedirect.com/, accessed on 9 September 2024), and the Web of Science—All Databases option (https://www-webofscience-com.uplib.idm.oclc.org/wos/alldb/basic-search, accessed on 9 September 2024). Grey literature and non-peer reviewed materials were not searched. The scope of the literature searches was restricted to papers written in the English language and released between January 2014 and February 2024.

### 2.3. Selection of Studies and Data Extraction

Two authors (NGK and TM) independently worked at each of the following four stages of retrieving, screening, and reviewing the studies included in this paper: (i) title identification; (ii) title and abstract screening; (iii) full-text retrieval and eligibility screening; and (iv) review of the eligible full texts and data extraction. The researchers’ disagreements were worked out through conversation until an agreement was reached.

Microsoft Excel^®^ (Microsoft Corporation, Redmond, WA, USA) and EndNote version 20 (Philadelphia, PA, USA) were used to manage the retrieved studies. Duplicate records were eliminated after compiling the search titles and abstracts from the three databases. Subsequently, the titles and abstracts underwent an eligibility screening, employing the following inclusion criteria: Peer-reviewed English articles (i) that examine the prevalence of any of the ESKAPE pathogens in food, animals, humans, and the environment; (ii) or quasi-cross-sectional studies (iii) that report on the total sample size, positive samples, and/or prevalence rates; and (iv) that report on studies conducted in African countries and published between January 2014 and February 2024. After screening the titles and abstracts, the full texts of the eligible studies were evaluated using the same criteria listed above.

Data extracted from eligible studies included the following: (a) author name, (b) year of publication, (c) location, (d) year of publication, (e) type of samples, (f) identification methods, and (g) positive samples of ESKAPE pathogen from humans, animals, and the environment.

### 2.4. Data Synthesis and Data Analysis

Comprehensive Meta-Analysis Software ver.4.0 program (https://www.meta-analysis.com/, accessed on 9 September 2024) was used to conduct the meta-analysis of ESKAPE pathogens on the humans, animals, environment, and food in the African continent. The PPE of each isolate was calculated, and subgroup analysis was performed according to continent, country, year of publication, settings, identification method, and samples collected. Random-effects models were used to generate forest plots showing the study-specific effect sizes with a 95% confidence interval (CI) for the PPE. The *I*^2^ statistic was used to measure heterogeneity among the studies. A value close to 0% indicated no heterogeneity, whereas a value close to 25%, 50%, and 75% corresponded to low, moderate, and high heterogeneity, respectively. The *p*-values corresponded to the heterogeneities between the studies from a Chi-squared test of the null hypothesis that there was no heterogeneity. Subgroup analysis was carried out on the study’s outcome based on ESKAPE pathogens, countries, study year, and methods. Subgroup analyses with less than 3 studies were not included in meta-analysis.

### 2.5. Quality Assessment of the Studies

Two authors (NGK and TM) independently evaluated the quality of the studies using the Joanna Briggs Institute (JBI) Critical Appraisal Tool for prevalence studies [18]. The JBI checklist for cross-sectional studies tool has ‘Yes’, ‘No’, ‘Unclear’, or ‘Not applicable’ question types, and the scores were assigned as 1 for ‘Yes’ and 0 for ‘No’. Studies were then classified as having low, medium, and high quality when the summated points became 0–4, 5–7, and 7–9, respectively. Discrepancies in the assessment between the two reviewers were discussed and resolved. Articles with high and moderate bias risks were excluded from this study, and only those with low bias risks were included.

## 3. Results

### 3.1. Search and Screening Results

The search process yielded a total of 202 studies. A total of 10 duplicate studies were removed, and 192 remained after study titles and abstracts were reviewed. A total of 172 full-text articles were assessed for eligibility, resulting in the further exclusion of 71 studies for various reasons (Figure 1). Meta-analysis was based on a total of 101 articles that reported the prevalence of ESKAPE pathogens. The quality assessment score from the Joanna Briggs Institute critical appraisal had an average of nine.

### 3.2. Overall Number of Reported ESKAPE Bacterial Isolates

Based on data sourced from included studies, the ESKAPE bacterial isolates reported by various studies on the African continent were *E. faecium* (*n* = 143 isolates), *S*. *aureus* (*n* = 3431), *K. pneumoniae* (*n* = 2441), *A. baumannii* (416), *Enterobacter* spp. (*n* = 359) and *P. aeruginosa* (*n* = 324).

### 3.3. Meta-Analysis of ESKAPE Pathogens in Humans

**Figure 2:**
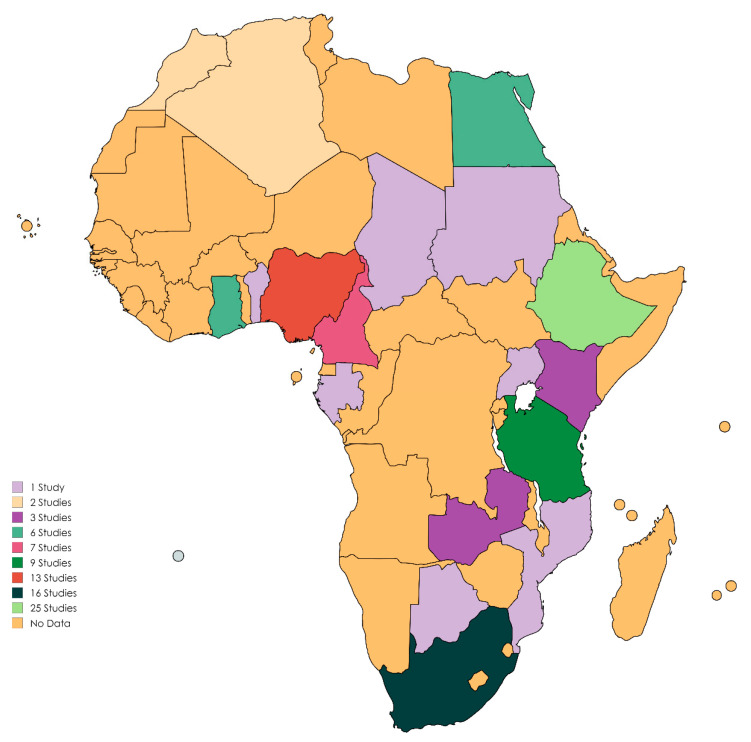
A map showing the number of studies included in this review per country in Africa [https://www.mapchart.net/world.html (accessed on 26 April 2024)]. A summary of studies from 2014 to 2024.

The continental prevalence of ESKAPE pathogens in humans was investigated in 54 studies (Supplementary Table S1) (Figure 2), of which the overall PPE of ESKAPE bacteria was 35.8% (95% CI: 29.8–42.4). Moreover, the species-specific PPE was 22.5% (95% CI: 17.1–28.9) for *S. aureus*, followed by *K. pneumoniae*, *P. aeruginosa*, *E. faecium*, *A. baumannii* and *Enterobacter* spp. with PPEs of 16.1% (95% CI: 10.9–23.3), 9.0% (95% CI: 3.4–17.7), 5.1% (95% CI: 1.3–17.5), 4.6% (95% CI: 1.6–12.4)], and 2.5% (95% CI: 1.3–4.8), respectively (Table 1).

The current study further showed that a higher number of studies (*n* = 31) were conducted between 2021 and 2024 with a PPE of 41.2% (95% CI: 31.9–51.2), while only 23 studies were conducted between 2010 and 2020 with a PPE of 28.9% (95% CI: 22.4–36.4). Moreover, studies that had multiple isolates from mixed samples (stool, urine and nasal swabs) had the highest number of ESKAPE positive isolates (*n* = 3536) with a PPE of 34.5% (95% CI: 26.5–43.4)]. The stool samples had a higher PPE of 44.0% (95% CI: 6.6–89.8) for ESKAPE bacterial isolates, followed by urine, nasal swabs, and blood samples, which were 37.3% (95% CI: 15.0–66.6), 26.9% (95% CI: 17.2–39.6), and 22.9% (95% CI: 17.1–29.9), respectively (Table 1).

In addition to the above-mentioned samples, the current study showed that culturing and biochemical testing were the most used techniques or methods for the detection of ESKAPE pathogens, with 3147 positive isolates. However, VITEK-MS had the highest PPE of 55.2% (95% CI: 32.2–76.1) as the most used methods for detecting and characterizing isolates, followed by whole genome sequencing (WGS) with a PPE of 37.1% (95% CI: 11.4–71.0), while PCR had a PPE of 33.2% (95% CI: 19.3–50.8). The PPE of MALDI-TOF MS was 28.4% (95% CI: 20.8–37.5), whilst culturing and biochemical testing had the lowest PPE of 29.1% (95% CI: 22.8–36.2) (Table 1).

**Figure 3:**
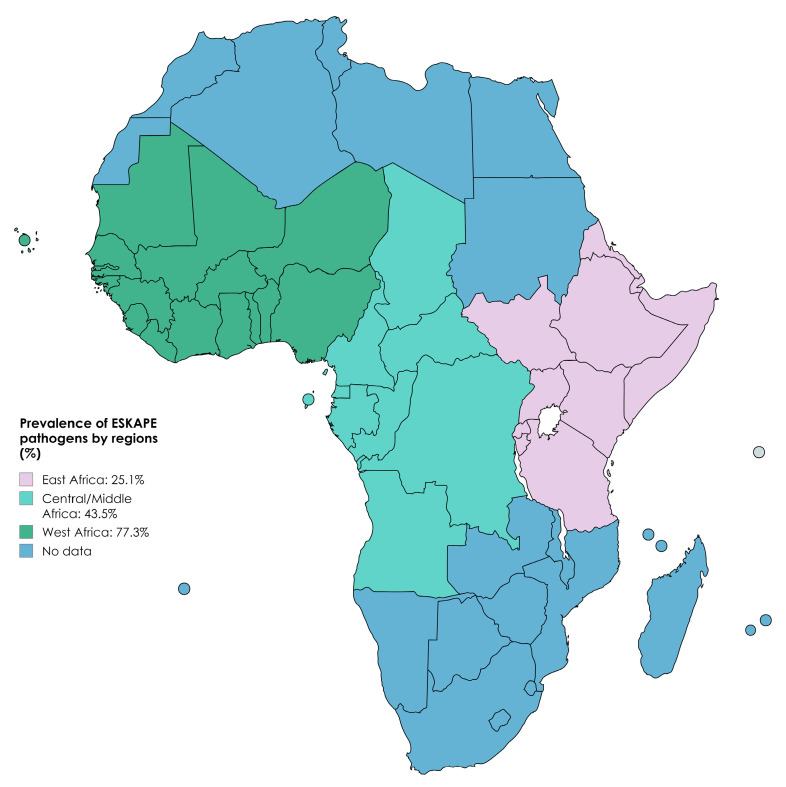
A map showing prevalence of ESKAPE pathogens by regions in Africa [https://www.mapchart.net/world.html (accessed on 28 August 2024)].

The current review showed that the highest PPE was recorded in West Africa with 77.3% (95% CI: 58.3–89.2), indicating that majority of the studies were reported in that region, followed by Central/Middle Africa and East Africa with 43.5% (95% CI: 23.9–65.4) and 25.1% (95% CI: 20.1–30.9), respectively (Figure 3).

Country analysis revealed that, in Ethiopia, *S. aureus* had highest PPE of detected ESKAPE pathogens with 21.5% (95% CI: 16.2–28.0), followed by *K. pneumoniae* with 12.5% (95% CI: 7.3–20.8) and *Enterobacter* spp. with 4.1% (95% CI: 2.2–7.5). Furthermore, the study showed that, in Nigeria, *S. aureus* had a PPE of 45.7% (95% CI: 21.7–71.9). Tanzania, on the other hand, had the highest PPE on *E. faecium* at 25.3% (95% CI: 14.8–39.8), followed by *A. baumannii* at 24.1% (95% CI: 8.1–53.3) and *K. pneumoniae* at 7.5% (3.9–13.8). While Cameroon had the highest PPE on *S. aureus* with 42.2% (95% CI: 21.4–66.1), followed by *K. pneumoniae* with 10.6% (4.3–23.9) (Table 1).

### 3.4. Meta-Analysis of ESKAPE Pathogens in Animals

The characteristics of all eligible animal studies included in this review are presented in Supplementary Table S2. Using a random-effect model, the pooled prevalence of ESKAPE pathogens isolated from animals was generated from two pathogens namely, *S. aureus* with 26.8% (95% CI: 15.3–42.7) and *Enterobacter* spp. with 10.5% (95% CI: 5.4–19.6). Polymerase chain reaction (PCR) was the most commonly used method to detect these pathogens with a PPE of 30.6% (95% CI: 16.1–50.4), while biochemical testing had the lowest PPE of 17.4% (95% CI: 7.6–34.9). 

Most of the ESKAPE bacteria were isolated from meat (beef, chicken, and mutton) and milk samples, resulting in PPEs of 41.7% (95% CI: 19.3–68.1) and 14.4% (95% CI: 6.7–28.2), respectively. Our year-based subgroup showed that more studies were conducted between 2010 and 2020 with a PPE of 70.4% (95% CI: 39.0–89.8), while between 2021 and 2024, the PPE was 16.6% (95% CI: 10.3–25.7). Our meta-analysis showed that South Africa was the only country where a PPE of 39.2% (95% CI: 24.5–56.2) for the detection of ESKAPE pathogens from animals could be generated (Table 2).

### 3.5. Meta-Analysis of ESKAPE Pathogens from the Environment 

For the environment, studies on the prevalence of ESKAPE pathogens were conducted in four countries (South Africa, Nigeria, Benin, Zambia, and Ethiopia) (Supplementary Table S3). The ESKAPE pathogens detected from the environment, namely *A. baumannii, S. aureus, K. pneumoniae*, and *Enterobacter* spp. had PPEs of 23.0% (95% CI: 12.9–37.8), 9.5% (95% CI: 2.6–29.4), 8.1% (95% CI: 1.3–37.4), and 7% (95% CI: 1–5.9), respectively.

Furthermore, in terms of methods used to isolate these pathogens, culturing and biochemical tests had the highest PPE of 44.5% (95% CI: 12.7–81.6), followed by PCR [38.3% (95% CI: 13.8–70.7)]. For the isolation and identification of ESKAPE pathogens per country, our meta-analysis generated a PPE of 33.8% (95% CI: 19.6–51.8) for South Africa because more studies were conducted in this country compared to other parts of the continent (Table 3).

### 3.6. Meta-Analysis of ESKAPE Pathogens from Food

The characteristics of all eligible food studies included in this review are presented in Supplementary Table S4. The overall PPE of ESKAPE pathogens from food sources was 34.2% (15, 7–59.4) (Table 4). Moreover, the study could only generate pooled prevalence estimates from the *S. aureus* [21.1% (95% CI: 13.4–31.4)] isolates, with PCR [38.5% (95% CI: 19.9–61.1)] as a method of identifying these pathogens, and from South Africa [57.6% (95% CI: 12.2–93.0)] because these were part of the subgroup analysis where more studies and data could be extracted in terms of the prevalence of ESKAPE pathogens from food sources.

### 3.7. Meta-Analysis of ESKAPE Pathogens from Humans and Animals

A total of eight studies were included in the overall analysis of the prevalence of ESKAPE pathogens from both humans and animals (Supplementary Table S5). The included studies were conducted in six countries, i.e., South Africa, Nigeria, Ghana, Zambia, Morocco, and Ethiopia. The overall pooled prevalence estimates of ESKAPE pathogens from both humans and animals were 18% (95% CI: 8.4–34.7) (Supplementary Table S5), with a prediction interval of 0.008–0.853, a Q value of 787.297, an *I^2^* of 99.111, a Q of 7, and a *p*-value of 0.000.

### 3.8. Meta-Analysis of ESKAPE Pathogens in Humans and the Environment

Three studies between Uganda, Ethiopia, and South Africa reported the prevalence of ESKAPE pathogens between humans and the environment (Supplementary Table S6). Their PPE was 41.6% (95% CI: 27.6–57.1), with a prediction interval of 0.001–0.99, a Q value of 24.4, an *I^2^* of 99.1, a Q of 7, and a *p*-value of 0.000.

### 3.9. Meta-Analysis of ESKAPE Pathogens in Humans, Animals, and the Environment

Lastly, the current study further revealed the PPE of 54.0% (95% CI: 30.5–75.8) between humans, animals, and the environment, with prediction intervals of 0.010–0.978, Q-value (151.3), df (Q) of 3, *p*-value of 0.000, and *I^2^* of 98.0.

### 3.10. Risk of Publication Bias of Included Studies

**Figure 4:**
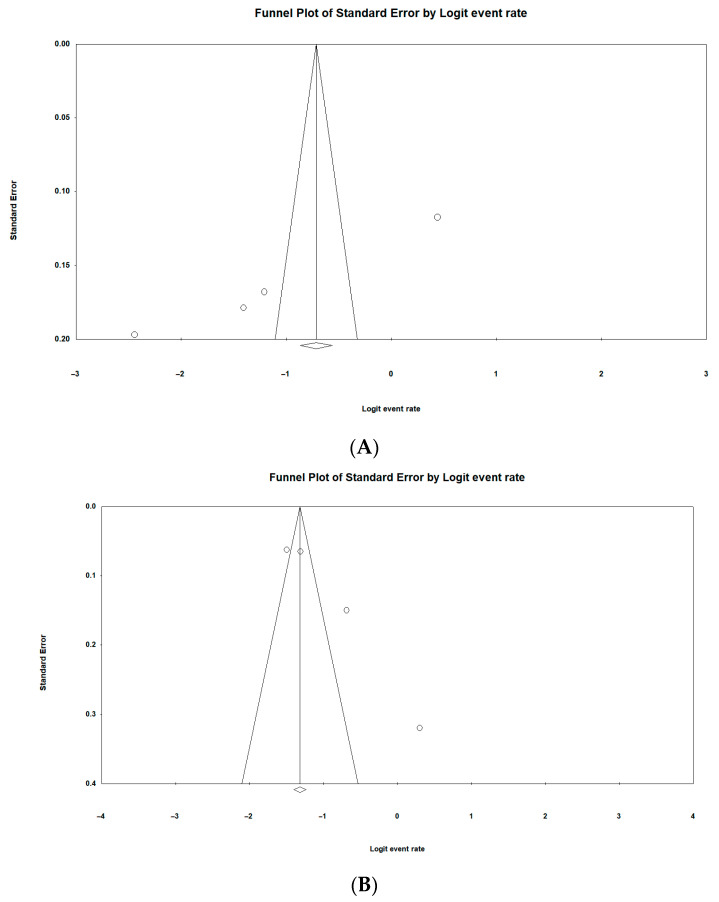

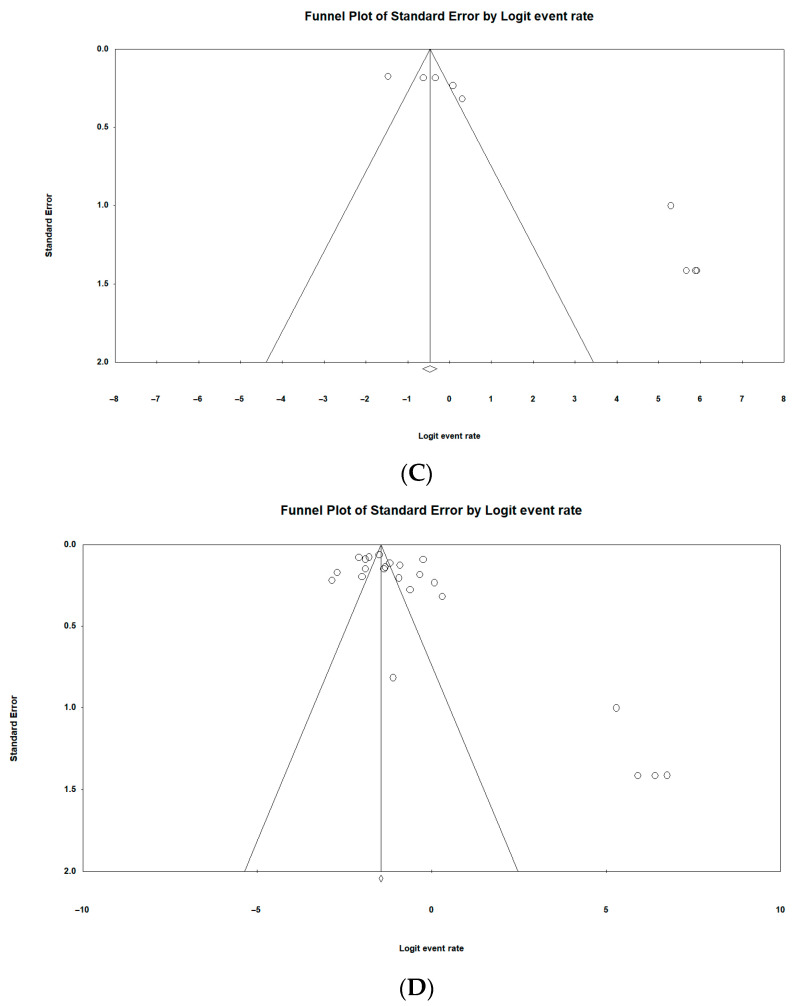
(**A**–**D**): Funnel plots indicating risk of publication bias from humans (**A**), animals (**B**), the environment (**C**) and food (**D**).

Publication bias was measured using funnel plots to test for symmetry, and this was further complemented using the Begg and Mazumdar rank correlation test and Egger’s regression test. The funnel plot was used to analyze the publication bias. Figure 4A–D display a practically symmetrical distribution of all the included studies on both sides of the funnel plot, indicating a relatively low potential for publication bias of the *p*-value < 0.04 for *A. baumannii*, *p*-value < 0.04 for MALDI-TOF MS, *p*-value < 0.01 for the West African region, and *p*-value < 0.01 for years (2010–2020), all in humans, respectively.

## 4. Discussion

Given their detrimental effects on the economy and overall mortality rate, the ESKAPE pathogens merit careful consideration in clinical settings as well as in research and development [19]. In this study, we thoroughly meta-analyzed the prevalence of ESKAPE bacteria reported in Africa from human, animal, and environmental sources, including food. The *S. aureus* was the most prevalent species (79.5%), followed by *A. baumannii* (27.6%), *K. pneumoniae* (24.2%), *Enterobacter* spp. (20%), *P. aeruginosa* (9.0%), and *E. faecium* (5.1%). A study on the ESKAPE pathogens in Italy conducted by Scaglione et al. [20] from 2015 to 2019 was not in agreement with our results as they discovered that *K. pneumoniae* was the most commonly occurring species (31.1%), followed by *P. aeruginosa* (19.8%), *S. aureus* (18.6%), *Enterobacter* spp. (13.4%), *A. baumannii* (13.2%), and *E. faecium* (3.8%). They were further supported by El-Kady et al. [21] in Saudi Arabia who discovered that *K. pneumoniae* was the most common species (47.4%), followed by *P. aeruginosa* (29.2%), *Enterobacter* spp. (14.0%), and *A. baumannii* (9.4%). The *A. baumannii* was the most common species (44%), followed by *S. aureus* (39%), *E. coli* (30%), *K. pneumoniae* (32%), and *Enterococcus* spp. (22%), in a study conducted by Ayobami et al. [22] between May 2016 and March 2017 in their global meta-analysis of the ESKAPE-E infections in low- and lower-middle-income countries. The aforementioned results show how these ESKAPE pathogens are distributed from one continent to the other.

*S. aureus* was also the most isolated pathogen with a PPE of 22.5%, followed by *K. pneumoniae*, *P. aeruginosa*, *E. faecium*, *A. baumannii*, and *Enterobacter* spp. with PPEs of 16.1%, 9.0%, 5.1%, 4.6%, and 2.5%, respectively, based on the prevalence of these pathogens on humans. The prevalence findings from our study are less than those of Schmidt et al. [23] and Iyamba et al. [24], who found the pathogen (*S. aureus*) in 83% and 56% of the Congo and South Africa, respectively. However, our results are in agreement with the findings of the study by Ojulong et al. [25] on the relative prevalence of MRSA in Uganda, where they found the species at 28.95%, which was significantly higher than the isolates of this pathogen found in Ethiopian hospitals by Dilnessa and Bitew [26] and Kahsay et al. [27]. In Hungary, Benko et al. [7] isolated this bacterium from the Emergency Department of a Tertiary Care Teaching Hospital with a prevalence rate of 11.3%, which is also lower than what our meta-analysis have revealed. Nirmal et al. [28] further recorded a prevalence of 12% in Intensive Care Units of a Tertiary Care Teaching Hospital in Delhi.

Moreover, Tenssaie [29] discovered *K. pneumoniae* in 22% of Ethiopian humans and 18.5% which is more than what our overall meta-analysis results show in Africa. Their results were further supported by various researchers who isolated this species at an alarming rate in Romania (60%) [30], Nigeria (>40%), and Ecuador (37%) [31]. According to Kerr and Snelling [32], *P. aeruginosa* is still a major nosocomial infection that is linked to morbidity and mortality, especially in immunocompromised people and fragile patients in intensive care units. Additionally, our meta-analysis revealed that the prevalence of *E. faecium* is 5.1%, which is less than the 23.5% identified by Ndubuisi et al. [33]. However, our findings are in line with those of Olawale, Fadiora, and Taiwo [34], who discovered that this species was 5.9% present in Nigeria. In addition, we discovered that the PPE of *Enterobacter* spp. in our study is 2.5%, significantly lower than the prevalence rate Falco et al. [35] reported in a Colombian study between 2011 and 2018, which revealed that 29% of patients were between the ages of 41 and 60 and 80% of KPC-producing *E. cloacae* complex were found in males. In a different study conducted in Iran between 2016 and 2018, 649 individuals had positive *Enterobacter* spp. results, of which 45.3% were female and 54.7% were male [36]. According to research conducted in a teaching hospital in China between 2015 and 2018, 62% of the patients with *E. cloacae* were men [37]. The findings of the study by Thu et al. [38] also demonstrated that Lao PDR had a substantially greater prevalence of *E. faecium* (65.7%) than in Thailand (47.1%).

Although the danger of zoonotic diseases to humans has not been well investigated, it may be less than that of the growing livestock-associated MRSA burden in several regions of Europe [39]. Thus far, the only indicator of potential future issues is the MRSA colonization in pigs which has been reported in South Africa (12.5%) and Senegal (1.3%). Data on a total of 2853 screened samples throughout Africa showed that 699 samples had *S. aureus* contamination. Raw, unprocessed beef, pork, goat, camel, lamb, and sheep meat, as well as unidentified red meat items, were among the positive samples. The *S. aureus* was found in meat products at a total prevalence of 24.5%. Of all red meats, samples from beef had the highest frequency, with 33.08% of the samples testing positive. According to these results, there may be a greater chance of human infections from beef [40]. The *S. aureus* was found in companion animals (13.6%) and veterinary hospital environments (7.1%), respectively. There is a higher rate of *S. aureus* colonization in the hospital environment (4.9%) and in companion animals (8.1%) compared to a previous Korean study on *S. aureus* in veterinary facilities.

Our meta-analysis was only able to generate a PPE (21.1%) for *S. aureus* isolation from the food items. Our findings closely resemble those of Mekhloufi et al. [41], who found this bacterium in foods in Algeria at a rate of 23.3%. In addition, Chaalal et al. [42] and Titouche et al. [43] revealed an overall *S. aureus* prevalence of 23.8% in the baked goods and cooked dishes taken from supermarkets and university cities in Western Algeria. In South Africa, ready-to-eat meat products were found to have a prevalence of 33.26% across all provinces [44]. As some authors [45] have pointed out, these variations are related to a number of factors, such as the source (shops or street vendors) and type of samples (foods derived from animals or not), the sample size, the accuracy of the identification method (based on cultivation characteristics, biochemical tests, or molecular biology techniques), the manufacturing procedures (whether or not bactericidal temperatures are used), and the general hygienic measures that are put in place during the preparation and handling of the foods. Specifically, *S. aureus*, a commensal bacteria found on human and animal mucosal membranes, skin, and nose [38], can contaminate food, particularly in situations where inadequate hygiene practices and conditions prevail. Remarkably, food handlers are identified as the primary source of food contamination with *S. aureus*, aside from contamination that may come from animals during the primary manufacturing stage [46,47].

## 5. Limitations

Variations in testing procedures and microbiological practices between laboratories may have resulted in incomplete data due to missing information not being recorded on the laboratory information system or non-standardized coding of the ESKAPE organisms. Moreover, there is no regular monitoring or surveillance of these pathogens in Africa, hence most studies tend to focus on one species such as *S. aureus*; however, for species such as *A. baumannii* and *Enterobacter* spp., studies are limited. 

## 6. Conclusions

Despite the differences in prevalence, there appears to be a similar incidence of ESKAPE bacteria reported from countries in Africa, Europe, North America, and Asia. To our knowledge, this was the first study to be conducted looking at the continental prevalence of ESKAPE pathogens in a One Health perspective. Our study has documented the separation of the ESKAPE bacteria from humans, animals, the environment and food, despite their predominance in nosocomial settings. Moreover, another big problem observed in this study was the distribution of the quality of the microbiological laboratory services from one country to the other; therefore, the responsible bodies should work on giving attention to practices to control the spread of these pathogens from one source to the other. Furthermore, our study will help to more accurately estimate the biological risks associated with using environmental waters (or other sources that are being investigated) for domestic and potable activities.

## Figures and Tables

**Table 1:** Subgroup analysis of ESKAPE pathogens in humans.

Risk Factors	Number of Studies	Pooled Estimates			Measure of Heterogeneity	
		Sample Size	ESKAPE Positive	*I*^2^ (95% CI)	Q Value	*I^2^*
**Overall**						
*S. aureus*	28	8804	1885	22.5% (17.1–28.9)	804.4	96.6
*K. pneumoniae*	24	12,292	1531	16.1% (10.9–23.3)	1248	98.1
*Enterobacter* spp.	12	7789	149	2.5% (1.3–4.8)	166.1	93.3
*P. aeruginosa*	7	1392	234	9.0% (3.4–17.7)	84.9	92.9
*E. faecium*	6	2004	143	5.1% (1.3–17.5)	228.4	97.8
*A. baumannii*	6	2774	92	4.6% (1.6–12.4)	115.0	95.6
**Study year**						
2010–2020	23	10,681	2812	28.9% (22.4–36.4)	675.2	96.7
2021–2024	31	13,006	3932	41.2% (31.9–51.2)	1725.1	99.2
**Samples**						
Mixed samples	28	12,650	3536	34.5% (26.5–43.4)	1370.9	98.0
Nasal Swabs	7	1930	518	26.9% (17.2–39.6)	130.5	95.4
Stool	4	2834	749	44.0% (6.6–89.8)	288.6	99.3
Blood	4	3786	711	22.9% (17.1–29.9)	46.3	93.5
Urine	3	709	353	37.3% (15.0–66.6)	67.8	97.0
**Methods**						
PCR	5	1321	462	33.2% (19.3–50.8)	141.8	97.1
Culturing, Biochemical	31	11,425	3147	29.1% (22.8–36.2)	981.5	96.9
VITEK-MS	6	3765	1160	55.2% (32.2–76.1)	512.8	99.0
WGS	3	2295	693	37.1% (11.4–71.0)	88.6	97.7
MALDI-TOF MS	4	3369	704	28.4% (20.8–37.5)	51.7	94.2
**Regions**						
East Africa	30	14,210	3858	25.1% (20.1–30.9)	1011.2	97.1
West Africa	9	1285	904	77.3% (58.3–89.2)	137.4	94.1
Central/Middle Africa	8	3239	1135	43.5% (23.9–65.4)	591.3	98.8
**Countries**						
**Ethiopia**						
*S. aureus*	9	3505	693	21.5% (16.2–28.0)	115.3	93.0
*K. pneumoniae*	6	4364	445	12.5% (7.3–20.8)	176.9	97.1
*Enterobacter* spp.	5	2436	84	4.1% (2.2–7.5)	25.0	88.0
**Nigeria**						
*S. aureus*	5	792	344	45.7% (21.7–71.9)	105.4	96.2
**Tanzania**						
*E. faecium*	7	3014	837	25.3% (14.8–39.8)	209.1	97.1
*K. pneumoniae*	5	2616	261	7.5% (3.9–13.8)	83.3	95.2
*A. baumannii*	4	1052	298	24.1% (8.1–53.3)	197.2	98.4
**Cameroon**						
*S. aureus*	3	632	275	42.2% (21.4–66.1)	65.6	96.9
*K. pneumoniae*	3	386	45	10.6% (4.3–23.9)	14.8	86.5

**Table 2:** Subgroup analysis of ESKAPE pathogens in animals.

Risk Factors	Number of Studies	Pooled Estimates			Measure of Heterogeneity	
		Sample Size	ESKAPE Positive	*I^2^* (95%CI)	Q Value	*I* ^2^
**Overall**						
*S. aureus*	7	2188	617	26.8% (15.3–42.7)	285.2	97.8
*Enterobacter* spp.	3	1908	181	10.5% (5.4–19.6)	22.7	91.2
**Methods**						
PCR	5	1586	514	30.6% (16.1–50.4)	203.8	99.0
Biochemical	3	2092	289	17.4% (7.6–34.9)	28.3	92.9
**Samples**						
Milk	3	2158	239	14.4% (6.7–28.2)	58.0	96.6
Meat (Beef, chicken and sheep)	4	1066	528	41.7% (19.3–68.1)	97.3	96.9
**Years**						
2010–2020	5	1313	658	70.4% (39.0–89.8)	201.8	98.01
2021–2024	7	3078	453	16.6% (10.3–25.7)	151.9	96.0
**Countries**						
South Africa	4	1015	428	39.2% (24.5–56.2)	78.3	96.1

**Table 3:** Subgroup analysis of ESKAPE pathogens from the environment.

Risk Factors	Number of Studies	Pooled Estimates			Measure of Heterogeneity	
		Sample Size	ESKAPE Positive	*I*^2^ (95%CI)	Q Value	*I^2^*
**Overall**						
*S. aureus*	4	1180	110	9.5% (2.6–29.4)	116.6	95.5
*K. pneumoniae*	3	910	37	8.1% (1.3–37.4)	45.0	95.5
*Enterobacter* spp.	3	903	29	7% (1.0–5.9)	35.8	94.4
*A. baumannii*	4	1698	324	23.0% (12.9–37.8)	36.8	91.8
**Methods**						
PCR	5	1044	221	38.3% (13.8–70.7)	99.3	95.9
Culturing, Biochemical	4	592	197	44.5% (12.7–81.6)	89.1	96.6
**Countries**						
South Africa	6	1851	394	33.8% (19.6–51.8)	64.7	92.2

**Table 4:** Subgroup analysis of ESKAPE pathogens in food.

Risk Factors	Number of Studies	Pooled Estimates			Measure of Heterogeneity	
		Sample Size	ESKAPE Positive	*I^2^* (95%CI)	Q Value	*I^2^*
**Overall**						
*S. aureus*	6	877	219	21.1% (13.4–31.4)	44.6	88.7
**Methods**						
PCR	5	867	569	38.5% (19.9–61.1)	78.4	94.8
**Countries**						
South Africa	3	515	462	57.6% (12.2–93.0)	50.1	96.0

## Data Availability

All data generated in this paper are included in the manuscript.

## Supplementary Materials

The following supporting information can be downloaded at: https://www.mdpi.com/article/10.3390/pathogens13090787/s1, Table S1: The continental prevalence of ESKAPE pathogens in humans; Table S2: The characteristics of all eligible animal studies included in this review; Table S3: Prevalence of ESKAPE pathogens from the environment; Table S4: The characteristics of all eligible food studies included in this review; Table S5: Studies included in the overall analysis of the prevalence of ESKAPE pathogens from humans and animals; Table S6: Prevalence of ESKAPE pathogens between humans and the environment.

## References

[1] Loyola-Cruz M.Á., Gonzalez-Avila L.U., Martínez-Trejo A., Saldaña-Padilla A., Hernández-Cortez C., Bello-López J.M., Castro-Escarpulli G. (2023). ESKAPE and beyond: The burden of coinfections in the COVID-19 pandemic. Pathogens.

[2] Catalano A., Iacopetta D., Ceramella J., Pellegrino M., Giuzio F., Marra M., Rosano C., Saturnino C., Sinicropi M.S., Aquaro S. (2023). Antibiotic-resistant ESKAPE pathogens and COVID-19: The pandemic beyond the pandemic. Viruses.

[3] Rice L.B. (2008). Federal funding for the study of antimicrobial resistance in nosocomial pathogens: No ESKAPE. J. Infect. Dis..

[4] Santajit S., Indrawattana N. (2016). Mechanisms of antimicrobial resistance in ESKAPE pathogens. BioMed Res. Int..

[5] Mulani M.S., Kamble E.E., Kumkar S.N., Tawre M.S., Pardesi K.R. (2019). Emerging strategies to combat ESKAPE pathogens in the era of antimicrobial resistance: A review. Front. Microbiol..

[6] World Health Organization (2017). WHO Publishes List of Bacteria for which New Antibiotics are Urgently Needed. https://www.who.int/news/item/27-02-2017-who-publishes-listof-bacteria-for-which-new-antibiotics-are-urgently-needed.

[7] Benkő R., Gajdács M., Matuz M., Bodó G., Lázár A., Hajdú E., Papfalvi E., Hannauer P., Erdélyi P., Pető Z. (2020). Prevalence and antibiotic resistance of ESKAPE pathogens isolated in the emergency department of a tertiary care teaching hospital in hungary: A 5-year retrospective survey. Antibiotics.

[8] Denissen J., Reyneke B., Waso-Reyneke M., Havenga B., Barnard T., Khan S., Khan W. (2022). Prevalence of ESKAPE pathogens in the environment: Antibiotic resistance status, community-acquired infection and risk to human health. Int. J. Hyg. Environ. Health.

[9] Stull J.W., Weese J.S. (2015). Hospital-associated infections in small animal practice. Vet. Clin. Small Anim. Pract..

[10] Walther B., Tedin K., Lübke-Becker A. (2017). Multidrug-resistant opportunistic pathogens challenging veterinary infection control. Vet. Microbiol..

[11] Hrenovic J., Durn G., Music M.S., Dekic S., Troskot-Corbic T., Skoric D. (2017). Extensively and multi drug-resistant Acinetobacter baumannii recovered from technosol at a dump site in Croatia. Sci. Total Environ..

[12] Amarasiri M., Sano D., Suzuki S. (2020). Understanding human health risks caused by antibiotic resistant bacteria (ARB) and antibiotic resistance genes (ARG) in water environments: Current knowledge and questions to be answered. Crit. Rev. Environ. Sci. Technol..

[13] Akanbi O.E., Njom H.A., Fri J., Otigbu A.C., Clarke A.M. (2017). Antimicrobial susceptibility of Staphylococcus aureus isolated from recreational waters and beach sand in Eastern Cape Province of South Africa. Int. J. Environ. Res. Public Health.

[14] Gwenzi W. (2020). Occurrence, behaviour, and human exposure pathways and health risks of toxic geogenic contaminants in serpentinitic ultramafic geological environments (SUGEs): A medical geology perspective. Sci. Total Environ..

[15] Ebomah K.E., Okoh A.I. (2020). An African perspective on the prevalence, fate and effects of carbapenem resistance genes in hospital effluents and wastewater treatment plant (WWTP) final effluents: A critical review. Heliyon.

[16] Verraes C., Van Boxstael S., Van Meervenne E., Van Coillie E., Butaye P., Catry B., De Schaetzen M.A., Van Huffel X., Imberechts H., Dierick K. (2013). Antimicrobial resistance in the food chain: A review. Int. J. Environ. Res. Public Health.

[17] Page M.J., McKenzie J.E., Bossuyt P.M., Boutron I., Hoffmann T.C., Mulrow C.D., Shamseer L., Tetzlaff J.M., Akl E.A., Brennan S.E. (2021). The PRISMA 2020 statement: An updated guideline for reporting systematic reviews. BMJ.

[18] Munn Z., Peters M.D., Stern C., Tufanaru C., McArthur A., Aromataris E. (2018). Systematic review or scoping review? Guidance for authors when choosing between a systematic or scoping review approach. BMC Med. Res. Methodol..

[19] Centers for Disease Control and Prevention (2019). Antibiotic Resistance Threats in the United States, 2019 (2019 AR Threats Report). https://www.cdc.gov/drugresistance/biggest-threats.html.

[20] Scaglione E., Mantova G., Caturano V., Fanasca L., Carraturo F., Farina F., Pagliarulo C., Vitiello M., Pagliuca C., Salvatore P. (2022). Molecular epidemiology of genital infections in Campania Region: A retrospective study. Diagnostics.

[21] El-Kady R., Karoma S., Al Atrouni A. (2022). Multidrug-Resistant Gram-Negative ESKAPE Pathogens from a Tertiary-Care Hospital: Prevalence and Risk Factors. Egypt. J. Med. Microbiol..

[22] Ayobami O., Brinkwirth S., Eckmanns T., Markwart R. (2022). Antibiotic resistance in hospital-acquired ESKAPE-E infections in low-and lower-middle-income countries: A systematic review and meta-analysis. Emerg. Microbes Infect..

[23] Schmidt T., Kock M.M., Ehlers M.M. (2017). Molecular characterization of Staphylococcus aureus isolated from bovine mastitis and close human contacts in South African dairy herds: Genetic diversity and inter-species host transmission. Front. Microbiol..

[24] Iyamba J.M., Wambale J.M., Lukukula C.M. (2014). High prevalence of methicillin resistant staphylococci strains isolated from surgical site infections in Kinshasa. Pan Afr. Med. J..

[25] Ojulong J., Mwambu T.P., Joloba M., Bwanga F., Kaddu-Mulindwa D.H. (2009). Relative prevalence of methicilline resistant Staphylococcus aureus and its susceptibility pattern in Mulago Hospital, Kampala, Uganda. Tanzan. J. Health Res..

[26] Dilnessa T., Bitew A. (2016). Prevalence and antimicrobial susceptibility pattern of methicillin resistant Staphylococcus aureus isolated from clinical samples at Yekatit 12 Hospital Medical College, Addis Ababa, Ethiopia. BMC Infect. Dis..

[27] Kahsay A.G., Hagos D.G., Abay G.K., Mezgebo T.A. (2018). Prevalence and antimicrobial susceptibility patterns of methicillin-resistant Staphylococcus aureus among janitors of Mekelle University, North Ethiopia. BMC Res. Notes.

[28] Nirmal K., Gupta P., Ahmad N., Nath S., Singh N.P., Das S. (2024). Bacteriological profile and their antimicrobial susceptibility pattern among clinical suspected adult septicemia admitted patients: A study from tertiary care and teaching hospital. East. J. Med. Sci..

[29] Tenssaie Z.W. (2001). Multiple antimicrobial resistance in gram negative bacilli isolated from clinical specimens, Jimma Hospital, southwest Ethiopia. Ethiop. Med. J..

[30] Penes N.O., Muntean A.A., Moisoiu A., Muntean M.M., Chirca A., Bogdan M.A., Popa M.I. (2017). An overview of resistance profiles ESKAPE pathogens from 2010–2015 in a tertiary respiratory center in Romania. Rom. J. Morphol. Embryol..

[31] Bastidas-Caldes C., Cisneros-Vásquez E., Zambrano A., Mosquera-Maza A., Calero-Cáceres W., Rey J., Yamamoto Y., Yamamoto M., Calvopiña M., de Waard J.H. (2023). Co-harboring of beta-lactamases and mcr-1 genes in Escherichia coli and Klebsiella pneumoniae from healthy carriers and backyard animals in rural communities in Ecuador. Antibiotics.

[32] Kerr K.G., Snelling A.M. (2009). Pseudomonas aeruginosa: A formidable and ever-present adversary. J. Hosp. Infect..

[33] Ndubuisi J.C., Olonitola O.S., Olayinka A.T., Jatau E.D., Iregbu K.C. (2017). Prevalence and antibiotics susceptibility profile of Enterococcus spp. Isolated from some hospitals in Abuja, Nigeria. Afr. J. Clin. Exp. Microbiol..

[34] Olawale K.O., Fadiora S.O., Taiwo S.S. (2011). Prevalence of hospital-acquired enterococci infections in two primary-care hospitals in osogbo, southwestern Nigeria. Afr. J. Infect. Dis..

[35] Falco A., Guerrero D., García I., Correa A., Rivera S., Olaya M.B., Aranaga C. (2021). Molecular characterization of KPC-2-Producing Enterobacter cloacae complex isolates from cali, Colombia. Antibiotics.

[36] Afshar Z.M., Miladi R., Janbakhsh A., Mansouri F., Sayad B., Vaziri S., Afsharian M., Zamanian M.H., Shirvani M., Yavari S. (2021). The Prevalence and Pattern of Enterobacter Antibiotic Resistance in the Patients Admitted to Imam Reza Hospital in Kermanshah, Iran (2016–2018). J. Kermanshah Univ. Med. Sci..

[37] Wang Y., Xiong Y., Wang Z., Zheng J., Xu G., Deng Q., Wen Z., Yu Z. (2021). Comparison of solithromycin with erythromycin in Enterococcus faecalis and Enterococcus faecium from China: Antibacterial activity, clonality, resistance mechanism, and inhibition of biofilm formation. J. Antibiot..

[38] Thu W.P., Sinwat N., Bitrus A.A., Angkittitrakul S., Prathan R., Chuanchuen R. (2019). Prevalence, antimicrobial resistance, virulence gene, and class 1 integrons of Enterococcus faecium and Enterococcus faecalis from pigs, pork and humans in Thai-Laos border provinces. J. Glob. Antimicrob. Resist..

[39] Lozano C., Gharsa H., Ben Slama K., Zarazaga M., Torres C. (2016). Staphylococcus aureus in animals and food: Methicillin resistance, prevalence and population structure. A review in the African continent. Microorganisms.

[40] Cuny C., Layer-Nicolaou F., Werner G., Witte W. (2024). A look at staphylococci from the one health perspective. Int. J. Med. Microbiol..

[41] Mekhloufi O.A., Chieffi D., Hammoudi A., Bensefia S.A., Fanelli F., Fusco V. (2021). Prevalence, enterotoxigenic potential and antimicrobial resistance of Staphylococcus aureus and methicillin-resistant Staphylococcus aureus (MRSA) isolated from Algerian ready to eat foods. Toxins.

[42] Chaalal W., Chaalal N., Bourafa N., Kihal M., Diene S.M., Rolain J.M. (2018). Characterization of Staphylococcus aureus isolated from food products in Western Algeria. Foodborne Pathog. Dis..

[43] Titouche Y., Houali K., Ruiz-Ripa L., Vingadassalon N., Nia Y., Fatihi A., Cauquil A., Bouchez P., Bouhier L., Torres C. (2020). Enterotoxin genes and antimicrobial resistance in Staphylococcus aureus isolated from food products in Algeria. J. Appl. Microbiol..

[44] Madoroba E., Magwedere K., Chaora N.S., Matle I., Muchadeyi F., Mathole M.A., Pierneef R. (2021). Microbial communities of meat and meat products: An exploratory analysis of the product quality and safety at selected enterprises in South Africa. Microorganisms.

[45] Abdeen E.E., Mousa W.S., Abdelsalam S.Y., Heikal H.S., Shawish R.R., Nooruzzaman M., Soliman M.M., Batiha G.E., Hamad A., Abdeen A. (2021). Prevalence and characterization of coagulase positive Staphylococci from food products and human specimens in Egypt. Antibiotics.

[46] Saad S.M., Hassanin F.S., Shaltout F.A., Nassif M.Z., Seif M.Z. (2019). Prevalence of methicillin-resistant Staphylococcus aureus in some ready-to-eat meat products. Am. J. Biomed. Sci. Res..

[47] Ire F., Imuh V. (2016). Bacteriological quality evaluation and safety of randomly selected ready-to-eat foods sold in Port Harcourt City, Nigeria. J. Appl. Life Sci. Int..

