# Supplementary material for: “One Health” Perspective on Prevalence of ESKAPE Pathogens in Africa: A Systematic Review and Meta-Analysis
Source: Pathogens. 2024 Sep 12;13(9):787. doi: 10.3390/pathogens13090787 (PMC11434769; doi:10.3390/pathogens13090787)
Supplement: Supplementary file 1 [file pathogens-13-00787-s001.zip › pathogens-3179961-supplementary.pdf]

"One Health" perspective on prevalence of ESKAPE pathogens in Africa: A systematic review and meta-analysis

Khasapane N.G.[1*], Nkhebenyane S.J.[1], Lekota K.[2], Thekisoe O.[2] , Ramatla T.[1,2]

Supplementary Table S1. Overview of ESKAPE pathogens isolated from humans in this study.

		E. faecium		***S. aureus***		***K. pneumoniae***		***A. baumannii***		***P. aeruginosa***		***Enterobacter* spp.**	
**Study (Citation)**	**Country**	**Total**	**Cases**	**Total**	**Cases**	**Total**	**Cases**	**Total**	**Cases**	**Total**	**Cases**	**Total**	**Cases**
Abaza et al., 2017	Egypt	1583	*	1583	*	1583	*	1583	*	1583	175	1583	*
Amare et al., 2022	Ethiopia	290	*	290	*	290	68	290	*	290	*	290	*
Ashagrie et al., 2021	Ethiopia	384	*	384	*	384	*	384	*	384	*	384	26
Ayeni et al., 2015	Nigeria	185	*	185	17	185	*	185	*	185	*	185	*
Getenet et al. 2019	Ethiopia	300	*	300	86	300	*	300	*	300	*	300	*
Dimani et al., 2023	Cameroon	185	*	185	*	185	11	185	*	185	*	185	2
Ezeamagu et al., 2018	Nigeria	120	*	120	50	120	*	120	*	120	*	120	*
Chirindze et al., 2019	Mozambique	275	*	275	*	275	21	275	*	275	*	275	*
Deyno et al., 2017	Ethiopia	117	*	117	33	117	*	117	*	117	*	117	*
Dilnessa and Bitew, 2016	Ethiopia	1360	*	1360	194	1360	*	1360	*	1360	*	1360	*
Kibwana et al., 2022	Tanzania	200	*	200	*	200	46	200	*	200	*	200	21
Toru et al., 2018	Ethiopia	403	22	403	*	403	*	403	*	403	*	403	*
Tola et al., 2021	Ethiopia	269	*	269	*	269	39	269	*	269	*	269	*
Legese et al., 2022	Ethiopia	1416	*	1416	*	1416	103	1416	*	1416	*	1416	27
Legese et al., 2018	Ethiopia	242	*	242	29	242	*	242	*	242	*	242	*

Mekonnen et al., 2021	Ethiopia	254	*	254	*	254	*	254	16	254	18	254	*
Moremi et al. 2019	Tanzania	1073	*	1073	140	1073	*	1073	*	1073	*	1073	*
Ndedy et al., 2023	Cameroon	264	*	264	164	264	*	264	*	264	*	264	*
Okamo et al., 2016	Tanzania	314	*	314	66	314	*	314	*	314	*	314	*
Olajuyigbe et al., 2017	Nigeria	200	*	200	199	200	*	200	*	200	*	200	*
Ong'era et al., 2023	Kenya	176	76	176	14	176	*	176	*	176	*	176	*
Reta et al., 2017	Ethiopia	400	*	400	52	400	*	400	*	400	*	400	*
Sangare et al., 2016	Mali	40	*	40	*	40	14	40	*	40	*	40	9
Abera et al., 2021	Ethiopia	403	11	*	*	*	*	*	*	*	*	*	*
Ajimuda et al., 2022	Nigeria	*	*	*	*	180	172	*	*	*	*	*	*
Alfeky et al., 2022	Egypt	*	*	*	170	*	*	*	*	*	*	*	*
Kejela and Dekosa, 2022	Ethiopia	*	*	384	126	*	*	*	*	*	*	*	*
Abera, e al., 2016	Ethiopia	*	*	*	*	477	49	*	*	*	*	210	8
Beyene et al., 2017	Ethiopia	*	*	8	2	*	*	*	*	*	*	*	*
Ndedy et al., 2022	Cameroon	264	*	264	70	264	*	264	*	264	*	264	*
Kumburu et al., 2018	Tanzania	*	*	575	33	*	*	*	*	*	*	*	*

Supplementary Table S2. Overview of ESKAPE pathogens isolated from animals in this study.

		E. faecium		***S. aureus***		***K. pneumoniae***		***A. baumannii***		***P. aeruginosa***		***Enterobacter* spp.**	
**Study (Citation)**	**Country**	**Total**	**Cases**	**Total**	**Cases**	**Total**	**Cases**	**Total**	**Cases**	**Total**	**Cases**	**Total**	**Cases**
Abdallah et al., 2022	Egypt	*	*	*	*	*	*	*	*	*	*	100	6
Abegewi et al., 2022	Cameroon	*	*	*	*	1608	15	*	*	*	*	1608	137
Yimana and Tesfaye, 2022	Ethiopia	*	*	384	37	*	*	*	*	*	*	*	*

Dweba, Zishiri and El Zowalaty, 2019	South Africa	*	*	403	217	*	*	*	*	*	*	*	*
Kissinga et al., 2018	Tanzania	100	100	*	*	*	*	*	*	*	*	*	*
Mkize, Zishiri and Mukaratirwa, 2017	South Africa	*	*	194	104	*	0	*	*	*	*	*	*
Mourabit et al., 2020	Morocco	*	*	421	42	*	0	*	*	*	*	*	*
Larbi et al., 2022	Ghana	*	*	*	*	200	3	*	*	*	*	200	38
Igbinosa et al., 2023	Nigeria	*	*	368	110	*	0	*	*	*	*	*	*
Wambui et al., 2018	Kenya	*	*	*	*	*	*	*	*	*	*	*	*
Wilson et al., 2024	South Africa	252	*	252	57	252	*	252	*	252	*	252	*
Khasapane et al., 2024	South Africa	166	*	166	50	166	*	166	*	166	*	166	*

Supplementary Table S3. Overview of ESKAPE pathogens isolated from the environment in this study.

		E. faecium		***S. aureus***		***K. pneumoniae***		***A. baumannii***		***P. aeruginosa***		***Enterobacter* spp.**	
**Study (Citation)**	**Country**	**Total**	**Cases**	**Total**	**Cases**	**Total**	**Cases**	**Total**	**Cases**	**Total**	**Cases**	**Total**	**Cases**
Adesoji et al., 2019	Nigeria	*	*	150	45	*	*	*	*	*	*	*	*
Akanbi et al., 2017	South Africa	*	*	245	30	*	*	*	*	*	*	*	*
Mousse et al., 2020	Benin	*	*	112	0	*	*	*	*	*	*	*	*
Anane et al., 2019	South Africa	*	*	*	*	*	*	1287	183	*	*	*	*
Eze, El Zowalaty and Pillay, 2021	South Africa	*	*	*	*	*	*	*	71	*	*	*	*
Mwanamoonga et al., 2023	Zambia	*	*	*	*	*	*	*140*	20	*	*	*	*
Mapipa et al., 2021	South Africa	*	*	*	*	*	*	*	*	36	54	*	*

Mwikuma et al., 2023	Zambia	153	16	*	*	*	*	*	*	*	*	*	*
Akinola, Okedigba and Elutade, 2022	Nigeria	*	*	56	30	*	*	*	*	*	*	*	*
Abera, Kibret, Mulu, 2016	Ethiopia	*	*	*	*	274	6	*	*	*	*	274	4
Adjei et al., 2021	South Africa	*	*	*	*	*	*	200	50	*	*	*	*
Ramsamy et al., 2021	South Africa	*	*	*	*	19	9	*	*	*	*	12	6
Odoyo et al., 2023	Kenya	617	2	617	5	617	22	617	23	617	2	617	19

Supplementary Table S4. Overview of ESKAPE pathogens isolated from the food in this study.

		E. faecium		***S. aureus***		***K. pneumoniae***		***A. baumannii***		***P. aeruginosa***		***Enterobacter* spp.**	
**Study (Citation)**	**Country**	**Total**	**Cases**	**Total**	**Cases**	**Total**	**Cases**	**Total**	**Cases**	**Total**	**Cases**	**Total**	**Cases**
Beshiru et al., 2023	Nigeria	*	*	*	*	360	6	*	*	*	*	*	*
Akindolire, Babalola and Ateba, 2015	South Africa	*	*	200	65	*	*	*	*	*	*	*	*
Ajuzieogu et al., 2022	Nigeria	*	*	10	3	10	8	*	*	*	*	10	3
Titouche et al., 2019	Algeria	*	*	270	62	*	*	*	*	*	*	*	*
Tshipamba et al., 2018	South Africa	115	3	115	5	*	*	*	*	*	*	*	*
Akindolire, Kumar and Ateba, 2018	South Africa	*	*	200	74	*	*	*	*	*	*	*	*
Chenouf et al., 2021	Algeria	*	*	82	10	*	*	*	*	*	*	*	*

Supplementary Table S5. Overview of ESKAPE pathogens isolated from humans and animals in this study.

Supplementary Table S6. Overview of ESKAPE pathogens isolated from humans and environment in this study

		E. faecium		*S. aureus*		*K. pneumoniae*		*A. baumannii*		*P. aeruginosa*		*Enterobacter* **spp.**	
**Study (Citation)**	**Country**	**Total**	**Cases**	**Total**	**Cases**	**Total**	**Cases**	**Total**	**Cases**	**Total**	**Cases**	**Total**	**Cases**
Egyir et al., 2020	Ghana	*	*	401	25	401	*	401	*	401	*	401	*
Mulemba et al., 2022	Zambia	*	*	598	198	598	*	598	*	598	*	598	
Mourabit et al., 2020	Morocco	*	*	497	72	497		497		497		497	
Egyir et al., 2022	Ghana	*	*	311	6	311	*	311	*	311	*	311	*
Schmidt, Kock and Ehlers, 2015	South Africa	*	*	3387	146	*	0	*	*	*	*	*	*
Banu and Geberemedhin, 2022	Ethiopia	311	*	311	52	311	*	311	*	311	*	311	*
Ngbede et al., 023	Nigeria	64	20	64	*	64	*	64	*	64	*	64	*
Jauro et al., 2022	Nigeria	200	*	200	101	200	*	200	*	200	*	200	*
Mayanja et al., 2023	Uganda	411	*	411	*	411	33	411	18	411	5	411	17
Abera et al., 2016	Ethiopia	274	*	274	*	274	49	274	*	274	*	274	8
Badul et al., 2021	South Africa	284	19	284	*	284	*	284	*	284	*	284	*
